# Activity of Membrane-Permeabilizing Lpt Peptides

**DOI:** 10.3390/biom14080994

**Published:** 2024-08-13

**Authors:** Stefano Maggi, Giulia Mori, Luigi Maglie, Dario Carnuccio, Danila Delfino, Emanuele Della Monica, Claudio Rivetti, Claudia Folli

**Affiliations:** 1 Department of Chemistry, Life Sciences and Environmental Sustainability, University of Parma, 43124 Parma, Italy; stefanomaggi1989@gmail.com (S.M.); giulia.mori@unipr.it (G.M.); luigi.maglie@unipr.it (L.M.); dario_carnuccio@libero.it (D.C.); emanuele.dellamonica@unipr.it (E.D.M.); 2 Department of Food and Drug, University of Parma, 43124 Parma, Italy; danila.delfino@unipr.it

**Keywords:** *Lacticaseibacillus*, toxin–antitoxin systems, type I toxins, membrane active peptides, membrane permeabilization, nucleoid condensation, liposome leakage

## Abstract

Herein, we investigated the toxicity and membrane-permeabilizing capabilities of Lpt and Lpt-like peptides, belonging to type I toxin–antitoxin systems carried by plasmid DNA of *Lacticaseibacillus* strains. These 29 amino acid peptides are predicted to form α-helical structures with a conserved central hydrophobic sequence and differently charged hydrophilic termini. Like Lpt, the expression of Lpt-like in *E. coli* induced growth arrest, nucleoid condensation, and cell membrane damage, suggesting membrane interaction as the mode of action. The membrane permeabilization activity of both peptides was evaluated by using liposome leakage assays, dynamic light scattering, and CD spectroscopy. Lpt and Lpt-like showed liposome leakage activity, which did not lead to liposome disruption but depended on peptide concentration. Lpt was generally more effective than Lpt-like, probably due to different physical chemical properties. Leakage was significantly reduced in larger liposomes and increased with negatively charged PCPS liposomes, indicating that electrostatic interactions and membrane curvature influence peptide activity. Contrary to most membrane-active peptides, Lpt an Lpt-like progressively lost their α-helical structure upon interaction with liposomes. Our data are inconsistent with the formation of membrane-spanning peptide pores but support a mechanism relying on the transient failure of the membrane permeability barrier possibly through the formation of “lipid pores”.

## 1. Introduction

Numerous membrane-permeabilizing peptides, many of which are associated with host defense activity, have been identified from various sources [1]. Several models have been proposed for membrane–peptide interaction, including the formation of trans-bilayer pores or channels, in which peptides align to form ion-channel-like structures, or induction of high curvature in the bilayer without specific peptide–peptide interactions [2,3]. Other models suggest that membrane-permeabilizing peptides do not form water-filled transmembrane pores but their action is explained by the carpet and the detergent models or by a more general interfacial activity model where peptides exert their effects primarily at the interface between the lipid bilayer of cell membranes and the aqueous environment [4,5,6,7]. *Lactobacillus* peptide toxins (Lpt) constitute a family of peptides that are part of the type I toxin–antitoxin (TA) systems Lpt/RNA, identified in the plasmid DNA of lactic acid bacteria [8,9]. Type I TA systems typically consist of a membrane-associated or cytosolic toxin peptide and an RNA antitoxin that inhibits toxin synthesis by interacting with its encoding mRNA [10]. Membrane-associated toxins have common features, such as small size less than 60 amino acids, a putative α-helical transmembrane domain with a hydrophobic core, and cationic residues generally localized at the C-terminal domain [10,11,12]. Their toxic activity is associated with membrane depolarization and/or permeabilization leading to intracellular ATP depletion [13]. The cytosolic type I toxins, such as RalR and SymE, are longer peptides or small proteins generally promoting nucleic acid cleavage [14,15]. These TA systems have been proposed to play diverse physiological roles, including plasmid maintenance, stress response, and antibiotic resistance [11,16].

The toxicity of the first identified Lpt peptide has previously been investigated in *E. coli* [8,17]. Cells transformed with an expression vector containing the Lpt coding sequence exhibited growth arrest upon induction of Lpt synthesis [18]. Fluorescence microscopy assays and AFM analysis revealed that the production of Lpt resulted in permeabilization of the *E. coli* membrane most probably through a carpeting mechanism, initially proposed to describe the mode of action of antibacterial peptides such as dermaseptin [19] and aurein [20]. Subsequent bioinformatics analysis identified three additional peptides (Lpt-like, Lpt-like1, and Lpt-like2), some occurring in multiple copies, with sequence identities ranging from 43% to 69% with Lpt, within the genomes of five *Lacticaseibacillus* strains [9]. Moreover, an extensive search across bacterial complete genomes archived in the NCBI database revealed five homologous peptides located on the plasmid DNA of *Lacticaseibacillus* strains [8]. With the aim to expand our knowledge about these TA systems, among these newly identified Lpt homolog peptides, we have chosen to characterize Lpt-like as one of the most represented within the *Lacticaseibacillus* plasmid DNA. In this study, we first conducted growth assays and fluorescence microscopy experiments to assess the toxicity of the Lpt-like peptide in *E. coli* cells. Secondly, we investigated the membrane-permeabilizing capabilities of both Lpt and Lpt-like peptides using liposome leakage assays, dynamic light scattering (DLS), and CD-spectroscopy.

## 2. Materials and Methods

### 2.1. Bioinformatics

Lpt and Lpt-like sequences were aligned using Clustal Omega at default settings at the EMBL-EBI webportal (https://www.ebi.ac.uk/jdispatcher/msa/clustalo (accessed on 5 June 2023)) [21]. The pairwise alignment figure was created with JalView v2.11.3.0 [22], using a user-defined color scheme. The isoelectric point of each sequence was calculated using the ProtParam tool (https://web.expasy.org/protparam/ (accessed on 12 October 2023)). Lpt and Lpt-like 3D structures were predicted using AlphaFold2 in ColabFold v1.5.5 (https://colab.research.google.com/github/sokrypton/ColabFold/blob/main/AlphaFold2.ipynb (accessed on 14 September 2023)) and represented using Pymol v2.5.5. Helical wheel projections were obtained with NetWheels web application (http://lbqp.unb.br/NetWheels/ (accessed on 2 July 2024)).

### 2.2. Lpt-like Cloning, Growth Assay, and Fluorescence Microscopy

Total DNA from *Lacticaseibacillus paracasei* 2306, available in the University of Parma Culture Collection (UPCC), was isolated as described in [9]. The Lpt-like coding sequence was amplified by PCR using primers Lpt-like_plus (CATATGAGTCCCTTCGATATTGCG) and Lpt-like_minus (GGATCCAACATCACTAAACCGTGTAGTC) and cloned into the NdeI/BamHI restriction sites of the inducible expression vector pET11b (Novagen) to obtain the recombinant plasmid pET11b-Lpt-like. The toxic activity of Lpt-like peptide was evaluated by analyzing the growth at 37 °C in LB medium of *E. coli* C41(DE3) pLysS cells (Lucigen, Middleton, UK) transformed with the inducible vector pET11b-Lpt-like by monitoring the OD at 600 nm in the presence and in the absence of 1 mM IPTG.

*E. coli* samples used for fluorescence microscopy analysis were prepared starting from single colonies of freshly transformed *E. coli* C41(DE3) pLysS cells grown overnight on solid media. After two hours of growth in liquid medium, the culture was split into two fractions, one of which was induced by the addition of 1 mM IPTG. In total, 1 mL of not-induced and induced cells were harvested after two hours, washed 3 times with 1 mL of PBS, and resuspended in 50 μL of PBS. DAPI and ethidium bromide dyes were added to a final concentration of 10 μg/mL each, followed by 5 min incubation at RT, washed twice with 1 mL of PBS to remove the excess of dye, resuspended in 50 μL of PBS, and imaged immediately. The glass coverslips were functionalized with poly-L ornithine (0.01%, Sigma-Aldrich, St. Louis, MO, USA) to favor cell adhesion. Fluorescence images were taken with a Nikon Eclipse E600 microscope equipped with a 100× oil immersion objective and with a Nikon DS-Fi2 digital camera. The UV-2A filter was used. The area of the nucleoid was measured using ad hoc written Matlab scripts as described in [17].

### 2.3. Liposome Preparation

A total of 200 µL of a solution of 25 mg/mL egg phosphatidylcholine (PC) in chloroform (Avanti Polar Lipids) was added to a glass vial and evaporated to dryness under a stream of nitrogen for 1 h. To ensure the complete removal of the solvent, the thin film was further dried under vacuum into an RVC2-18 (Christ) speed vac connected to a MZ2C (Vacuubrand, Wertheim, Germany) vacuum pump for 1 h at 25 °C. The dried lipid film was redissolved in 1 mL of 50 mM carboxyfluorescein (CF) (Sigma-Aldrich) in 10 mM HEPES, pH 7.4, 150 mM NaCl and heated in a water bath at 37 °C for 1 h, followed by three freezing and thawing cycles between liquid nitrogen (1 min) and 37 °C (1 min) to induce liposome formation. The suspension was collected in a Hamilton syringe and extruded 25 times through polycarbonate filters of 100 or 1000 nm pore sizes using a Mini-Extruder (Avanti Polar Lipids). The extruded sample was applied to a gel-filtration chromatography column of Sephadex G-50 and eluted with 10 mM HEPES pH 7.4 to separate CF-encapsulated liposomes from free CF. Additionally, liposomes were prepared using a combination of PC and porcine brain phosphatidylserine (PS; Avanti Polar Lipids) at a ratio of 80:20 or 50:50. Liposomes were also prepared without the inclusion of the fluorophore; in this case, the procedure did not involve gel filtration. Liposome size was verified by DLS as described in Section 2.8 of Materials and Methods. Liposomes were stored at 4 °C in the dark for up to two weeks.

### 2.4. Lipid Quantification

Liposome concentrations throughout the text represent the concentration of lipids determined by using the ammonium ferrothiocyanate method as described in [23]. Lipid extraction was carried out by adding an aliquot of the liposome sample to a vial containing 1 mL of chloroform and 1 mL of ammonium ferrothiocyanate. The mixture was then vortexed for 1 min to facilitate the extraction of lipids into the organic phase. After allowing the phases to separate, the upper aqueous phase containing the excess ammonium ferrothiocyanate was carefully removed and the absorbance of the bottom organic phase was measured at 488 nm using a spectrophotometer. The concentration of lipids was calculated from the absorbance at 488 nm using a regression equation and a correlation coefficient obtained experimentally from a standard curve obtained by using standard samples at increasing phospholipid concentrations (0.01–1 mg/mL).

### 2.5. Leakage Experiments

Freeze-dried Lpt and Lpt-like were purchased from CRIBI peptide facility (University of Padua) with a purity ≥ 95% as assessed by mass spectrometry and solubilized in 100% methanol. Leakage kinetics were performed by following the dequenching of CF fluorescence at 512 nm (excitation at 492 nm) using a spectrofluorometer LS 50B (Perkin Elmer) with a time step of 0.02 min. In a typical experiment, 36 μM liposomes in 10 mM HEPES pH 7.4 and increasing concentrations of Lpt peptides (0.7, 1, 2, and 4 μM) were used. CF encapsulation was verified by adding 0.1% Triton X-100 to the liposome sample and monitoring the increase in fluorescence intensity over a few minutes. Leakage assays were conducted as follows: first, baseline fluorescence intensity was recorded for several minutes on the liposome sample. Then, the peptide was added to the sample and fluorescence intensity was monitored until a plateau was reached. Finally, 0.1% Triton X-100 was added to completely dissolve the liposomes and the fluorescence intensity was recorded for a few minutes. The leakage percentage was calculated with the following equation [24]:

(1) $$\%Leakage\left(t\right)=100\left[I\left(t\right)-I_{0}\right]/\left[I_{max}-I_{0}\right]$$

where *I*(*t*) was fluorescence intensity at time t, *I*_0_ was the initial fluorescence intensity at the point at which peptide was added, and *I*_max_ was the maximum intensity reached after Triton X-100 addition. All experiments were conducted in triplicate and the mean values with SE were reported.

### 2.6. Hemolysis Experiments

Bovine blood was centrifuged at 15,000× *g* for 15 min and the insoluble fraction containing erythrocytes was resuspended in PBS buffer and centrifuged again 5 times. At the end, the erythrocytes were resuspended at 2.5% *v*/*v* in PBS buffer. For lysis assays, Lpt peptides dissolved in methanol were added to 100 μL of erythrocyte (0.25% *v*/*v* in PBS) to a final concentration of 1, 5, and 10 μM; the solution was incubated for 30 min at RT. As a control, the hemolysis activity of methanol was assessed. An aliquot of erythrocyte was analyzed in the presence of 3.3% methanol, which corresponds to the final concentration of methanol in the assay with 10 μM Lpt peptides. Additionally, samples containing only erythrocytes (negative control, no hemolysis) or erythrocytes with the addition of 1% Triton-X (positive control, complete hemolysis) were incubated under the same conditions. To evaluate the fraction of hemolysis, samples were centrifuged at 2000× *g* for 5 min and the absorbance spectra (350–650 nm) of supernatants were recorded by using a Jasco V-750 spectrophotometer. The OD at 414 nm, corresponding to the Soret peak of the porphyrin, was used to calculate the percentage of hemolysis with the equation:

(2) $$\%Hemolysis=100\left[{\mathrm{OD}}_{peptide}-{\mathrm{OD}}_{untreated}\right]/\left[{\mathrm{OD}}_{Triton\;X-100}-{\mathrm{OD}}_{untreated}\right]$$

where OD*_peptide_* is the OD at 414 nm, measured after incubation with Lpt peptides; OD*_untreated_* is the OD at 414 nm, measured for erythrocytes alone; and OD*_Triton X_*_-100_ is the OD at 414 nm, measured after the addition of Triton-X (100% hemolysis). Experiments were conducted in triplicate.

### 2.7. CD Spectroscopy

Far-UV CD spectra (200–240 nm) were recorded with a Jasco J1500 spectropolarimeter (Applied Photophysics Ltd., Surrey, UK) using a 0.1 cm path length cuvette, a scanning rate of 50 nm/min, and a bandwidth of 1.0 nm. For data collection, CD spectra were accumulated three times. CD spectra of Lpt peptides were recorded at a concentration of 100 μM in 10 mM sodium phosphate buffer pH 7.4. To evaluate peptide secondary structure stability, CD spectra were recorded at increasing temperature from 25 to 90 °C after 5 min incubation. To evaluate peptide secondary structure in the presence of lipids, CD spectra were recorded at 25 °C at increasing concentrations of 100 nm PC or PCPS liposomes. As a control, CD spectra of DksA (a small α-helical protein) 20 μM in 10 mM sodium phosphate buffer pH 7.4 were recorded in the absence and in the presence of 100 nm PC liposomes.

### 2.8. DLS Experiments

To confirm the dimension of the extruded vesicles, liposomes were subjected to dynamic light scattering (DLS) using a Malvern Zetasizer NANO ZSP (Malvern Panalytical, Malvern, UK). Solutions containing 36 μM PC or PCPS liposomes were treated with 4 μM Lpt or Lpt-like and incubated at RT for 1 h. Control samples of 36 μM PC or PCPS liposomes untreated or treated with 0.1% Triton X-100 or 4% methanol (the same concentration reached with the addition of the Lpt peptides) were also incubated at RT for 1 h. DLS measurements were performed at 25 °C. Data were analyzed with the Malvern Zetasizer software v7.13 using standard settings.

## 3. Results

### 3.1. Structural Comparison between Lpt and Lpt-like

**Figure 1:**
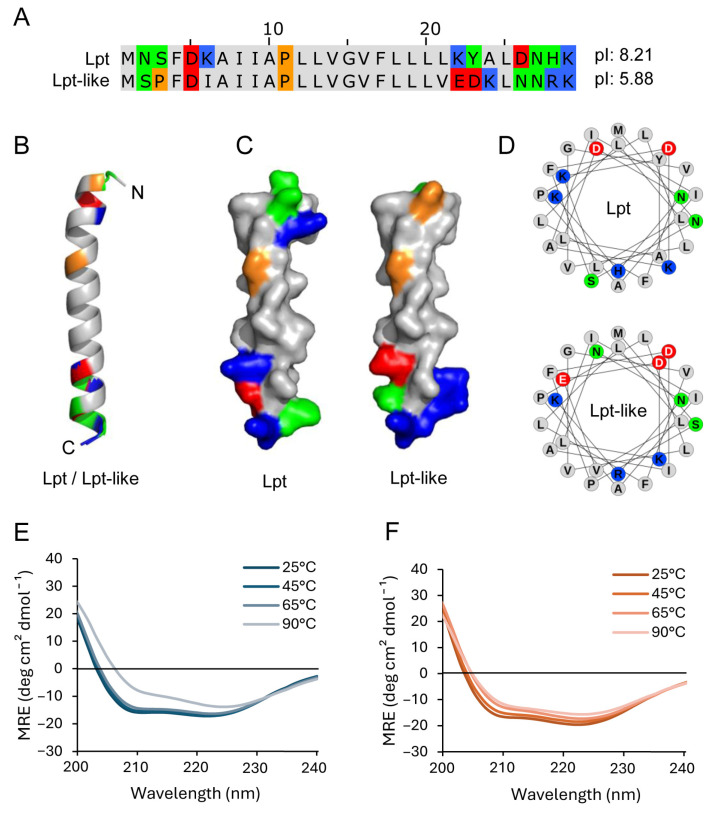
Structural characterization of Lpt peptides. (**A**) Sequence alignment of Lpt and Lpt-like. (**B**) Structure alignment of Lpt and Lpt-like in cartoon representation. (**C**) Structure of Lpt (**left**) and Lpt-like (**right**) in surface representation. (**D**) Helical wheel projection of Lpt and Lpt-like peptides. In (**A**–**D**), amino acids are colored according to their physical–chemical properties (hydrophobic: gray, hydrophilic: green, acidic: red, basic: blue, proline: orange). (**E**) CD spectra of Lpt 100 μM at increasing temperature. (**F**) CD spectra of Lpt-like 100 μM at increasing temperature.

In previous studies, we have identified several Lpt/RNA type I TA systems in *Lacticaseibacillus* strains [8,9] and analyzed the toxicity of the Lpt peptide in *E. coli* cells [8,17]. Among these peptides, Lpt-like has been found in four different reported plasmids and in the genome of three different strains. The sequence alignment and the structural models of Lpt and Lpt-like (Figure 1A–D) show a central hydrophobic sequence (AIIAPLLVGVFLLL), which is conserved in both peptides and N- and C-terminal regions characterized by the presence of polar amino acids. In the N-terminal sequence, a lysine residue present in Lpt is replaced by an isoleucine in Lpt-like, increasing its hydrophobicity, while, in the C-terminal region, Lpt-like contains a higher number of charged residues compared to Lpt. Furthermore, an additional proline residue is present in position 3 of Ltp-like, increasing the local structural rigidity. Thus, the predicted isoelectric points are 8.21 and 5.88 for Lpt and Lpt-like, respectively.

The CD spectra of both Lpt and Lpt-like at a concentration of 100 μM in sodium phosphate buffer pH 7.4 recorded at 25 °C show the minima at 208 nm and 222 nm typical of the α-helix (Figure 1E,F). In the case of Lpt, the increase in temperature up to 65 °C does not significantly change the CD spectra, while, at a temperature of 90 °C, there is a partial loss of the α-helix structure. In the case of Lpt-like, we observed a slight change in the CD spectra up to 90 °C, indicating a more stable α-helix structure.

### 3.2. Toxicity of Lpt-like Peptide in E. coli

**Figure 2:**
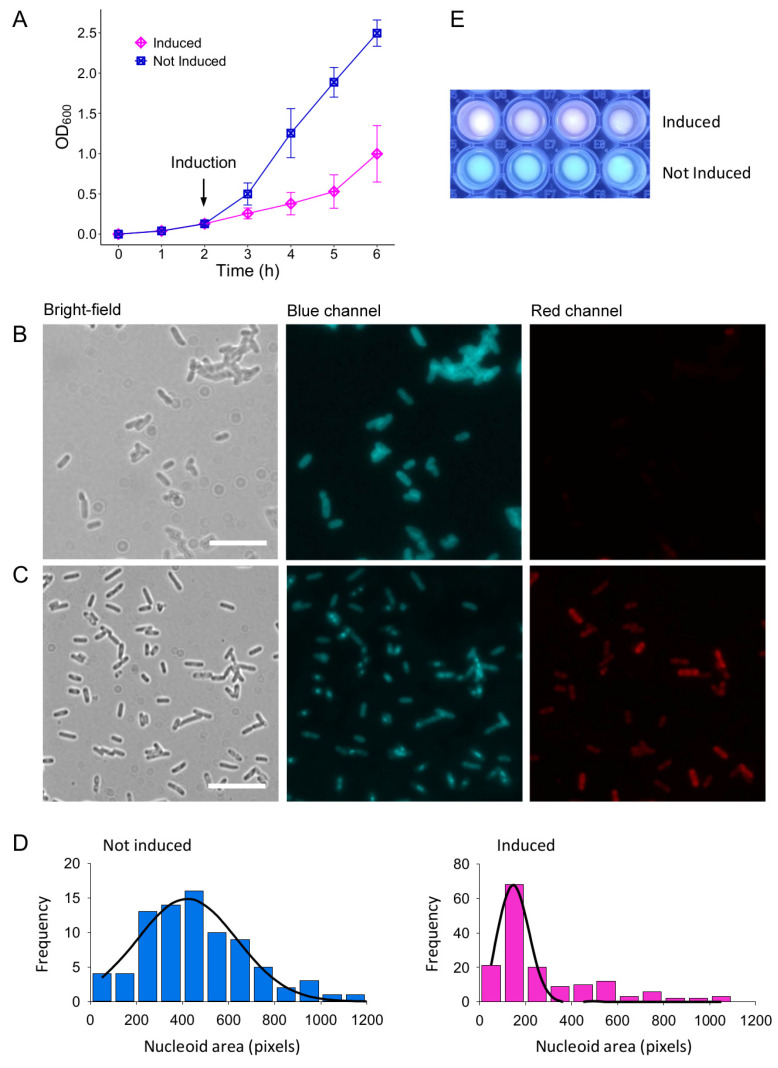
Effect of Lpt-like expression on *E. coli* cells. (**A**) Growth curve of *E. coli* C41 (DE3) pLySs cells transformed with the recombinant vector pET11b-Lpt-like and cultured at 37 °C without (not induced, blue) or with (induced, magenta) the addition of 1 mM IPTG after 2 h of growth. (**B**,**C**) Representative bright-field, blue channel and red channel of fluorescence images captured with noninduced (**B**) and induced (**C**) *E. coli* cells stained with DAPI and ethidium bromide. Size bar 10 μm. (**D**) Histogram plots showing the distribution of nucleoid areas of not-induced cells (blue) and induced cells (magenta). (**E**) UV transilluminator picture of wells containing induced or not-induced cells stained with DAPI and ethidium bromide.

The toxicity of the Lpt peptide was previously verified in *E. coli* C41(DE3) pLysS cells transformed with the recombinant vector pET11b-Lpt harboring the Lpt coding sequence under the control of the T7 promoter [17]. To validate the predicted toxicity of Lpt-like peptide, we have cloned the Lpt-like coding sequence of *L. paracasei* 2306 into pET11b to obtain pET11b-Lpt-like that was used to perform toxicity assay using the *E. coli* strain as above. Figure 2A shows that the growth of *E. coli* cells is significantly inhibited after IPTG induction, confirming the toxic activity of the Lpt-like peptide. However, after three hours from induction the growth rate of *E. coli* cells increases again, an effect also observed with Lpt and other type I toxins [17,25].

To characterize the morphology of Lpt-like peptide-expressing *E. coli* C41pLysS cells and to compare it with the phenotype of Lpt-overexpressing cells, we have employed fluorescence microscopy and the membrane-permeable blue fluorescent dye 4′,6-diamidino-2-phenylindole (DAPI), which preferentially stains the nucleoid DNA. For this analysis, cells were harvested after two hours of induction and compared with not-induced cells grown for the same time. Figure 2B depicts noninduced cells showing an elongated nucleoid that occupies the entire cell volume (blue channel). Conversely, in the case of induced cells, the DAPI fluorescence signal reveals a circular and more compact nucleoid located in the middle of the cell (Figure 2C, blue channel). To quantitatively analyze the observed nucleoid compaction of Lpt-like-expressing cells, the area of the blue-stained nucleoid was measured as described in Materials and Methods. As shown in Figure 2D, the mean of the distribution of the nucleoid area shifts from 420 ± 216 pixels to 150 ± 65 pixels upon IPTG induction. This result confirms that the bacterial nucleoid undergoes a significant condensation upon Lpt-like expression, in accordance with what was observed with Lpt [17].

To investigate whether Lpt-like expression similarly impacts membrane integrity as previously observed for Lpt, we conducted a series of experiments employing a mixture of DAPI and ethidium bromide (EtBr) staining. While DAPI stains all bacteria, the membrane-impermeable fluorescent dye EtBr permeates only de-energized bacteria [26] or bacteria with damaged membranes [27]. Aliquots of cells after two hours from induction and not-induced cells grown for the same time were stained with both DAPI and EtBr and analyzed by fluorescence microscopy as described in Materials and Methods (Figure 2B,C red channel). In the microscope view, cells exhibiting any degree of red fluorescence are classified as red, indicating a loss of membrane integrity. Under these conditions, out of a total of 665 induced cells, 83.3% displayed red fluorescence, while the remaining 16.7% appeared blue. Conversely, with uninduced cells, out of a total of 689 cells, only 4.4% exhibited red fluorescence, while the majority, 95.6%, appeared blue. The distinctive blue–red fluorescence signal emitted by the mixture of DAPI and EtBr, indicative of membrane damage caused by Lpt-like, was also evident on plates when examined using a UV transilluminator (λ_max_ 312 nm) (Figure 2E). These findings provide evidence that the expression of the Lpt-like peptide exerts a significant influence on membrane integrity, mirroring observations previously made for Lpt.

### 3.3. Liposome Leakage Assays

**Figure 3:**
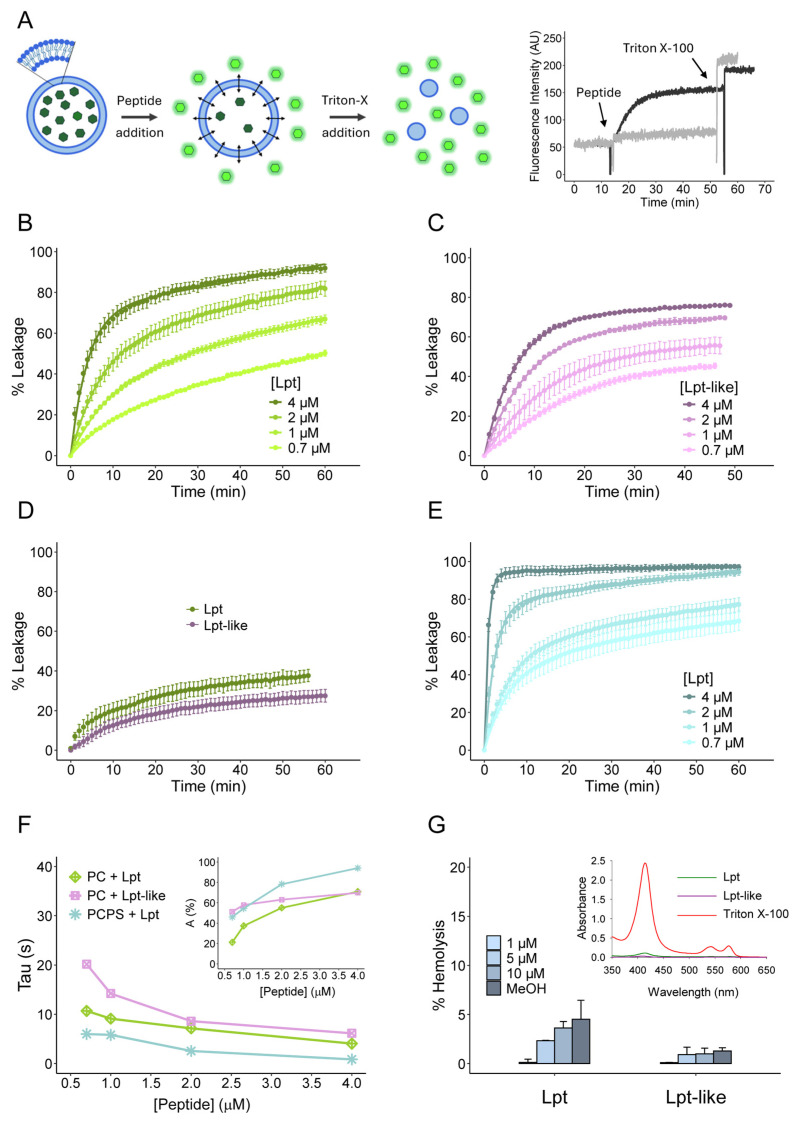
Membrane permeabilization activity of Lpt and Lpt-like. (**A**) Scheme and leakage assays with 36 μM 100 nm PC liposomes loaded with 50 mM CF in the presence and in the absence of Lpt peptide. At 12 min, 4 μM Lpt solubilized in 100% methanol (black line) or an equal volume of methanol (gray line) was added. At 55 min, Triton X-100 was added to both samples to completely disrupt the liposome. (**B**,**C**) Kinetics of CF leakage from 100 nm PC liposomes induced by Lpt (**B**) and Lpt-like (**C**) at concentrations ranging from 0.7 to 4 μM. (**D**) Kinetics of CF leakage from 1 μm PC liposomes by 4 μM Lpt or Lpt-like. (**E**) Kinetics of CF leakage from 100 nm PCPS (80:20) liposomes induced by Lpt at concentrations ranging from 0.7 to 4 μM. Data points of leakage experiments (**B**–**E**) represent the mean of three independent experiments with SE bars. (**F**) Comparison of Tau values for leakage experiments with 100 nm PC or PCPS (80:20) liposomes incubated with different concentrations of Lpt peptides. The corresponding A values are plotted in the inset. (**G**) Hemolysis assay using bovine red blood cells incubated with 1, 5, and 10 μM Lpt or Lpt-like solubilized in methanol and 3.3% methanol. Bars represent the mean percentage hemolysis of three independent experiments with SD. Inset: absorption spectra of the red blood cell supernatant after incubation with Lpt, Lpt-like, and 1% Triton X-100.

Liposomes are spherical phospholipid bilayers that can compartmentalize internal aqueous content from the external environment, making them a valuable tool in membrane integrity assays. Several fluorescent probes can be encapsulated, resulting in fluorescence quenching due to the high concentration of the fluorophore (Figure 3A). Leakage induced by membrane-permeabilizing peptides leads to the release of the fluorophore with an increase in the fluorescence signal (Figure 3A). To characterize the membrane-permeabilizing activity of Lpt and Lpt-like, we conducted leakage assays using LUV liposomes assembled in the presence of 50 mM carboxyfluorescein (CF) [28]. First, leakage kinetics were recorded using phosphatidylcholine (PC) liposomes with a diameter of 100 nm, resuspended in PBS buffer at a concentration of 36 μM, after the addition of increasing concentrations of the Lpt peptides (from 0.7 to 4 μM). As shown in Figure 3B,C, at all peptide concentrations tested, the leakage percentage increased rapidly within the first 10 min, followed by a slow linear increase over extended times.

The leakage kinetics recorded with different Lpt concentrations indicate that the initial velocity of membrane permeabilization increases with the peptide concentration. The same behavior was observed with Lpt-like; however, at the same peptide concentration, both the initial velocity and the percentage of leakage after 45 min were lower. For kinetic analyses, the time dependences of leakage were fitted to the following equation:

(3) $$\%Leakage=A\left(1-e^{-\left(\frac{time}{\tau }\right)}\right)+m\times time$$

where *A* is the size of the exponential component (the maximum percentage of leakage reached in the exponential phase), *τ* is the time constant for the exponential component (the *time* to reach leakage percentage *A* with the initial velocity), and *m* is the slope of the linear component [29]. *τ* and *A* values for Lpt and Lpt-like at different concentrations are reported in Figure 3F. The graphs show a difference in the permeabilization kinetics of Lpt and Lpt-like only at the lower concentrations.

To verify whether the membrane permeabilization activity of Lpt peptides was dependent on the liposome dimensions, the same experiments were repeated by using PC liposomes with a diameter of 1 μm (Figure S1). As shown in Figure 3D, the membrane permeabilization activity of either Lpt or Lpt-like at their highest tested concentration is significantly reduced. This effect may be attributed to the different membrane curvature/tension between liposomes of different sizes, as previously postulated [30]. Furthermore, membrane permeabilization activity of Lpt has also been evaluated using 100 nm PCPS (80:20) liposomes at a concentration of 36 μM. The leakage kinetics at increasing concentrations of Lpt peptide (from 0.7 to 4 μM) are reported in Figure 3E. When Lpt was added at the highest concentration (4 μM), the leakage percentage reached a value of 100% after 5 min. By fitting experimental data by using Equation (3), we calculated the *τ* and A values for each Lpt concentration (Figure 3F). In this case, *τ* values were lower than those determined for PC liposomes when incubated with both Lpt and Lpt-like. Accordingly, leakage kinetics carried out with Lpt and PCPS liposomes show A values higher than those obtained with PC liposome treated with both Lpt and Lpt-like (Figure 3F). These results suggest that the presence of phosphatidylserine, which adds an additional negative charge on the surface of the liposomes, favors the interaction with Lpt, which has an overall positive charge, thus leading to a more rapid permeabilization.

We have also verified the peptide leakage activity of Lpt peptides on a physiological membrane by using bovine erythrocytes. Membrane permeabilization was monitored spectrophotometrically by measuring the hemoglobin (Hb) release after incubation with Lpt peptides (Materials and Methods). As shown in Figure 3G, the hemolysis due to the addition of the Lpt peptides was more limited than that resulting from the addition of 3.3% methanol (<5% hemolysis). This result may be ascribed to the different membrane composition and the large size of erythrocytes (average diameter ~5 µm). In any case, this result indicates that the Lpt peptides do not disrupt the lipid bilayer in a way that allows the release of the Hb protein probe.

### 3.4. Lpt and Lpt-like Do Not Disrupt Liposomes

**Figure 4:**
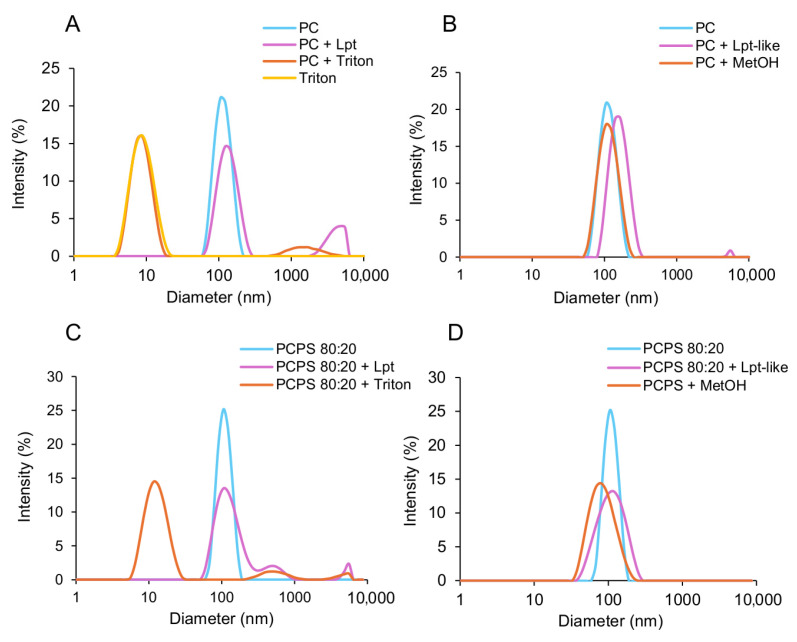
DLS size distributions of liposomes. (**A**) Size distributions of PC liposomes untreated, treated with Lpt, or treated with Triton X-100 0.1%, and of Triton X-100 0.1% alone. (**B**) Size distributions of PC liposomes untreated, treated with Lpt-like, or with methanol 4%. (**C**) Size distribution of PCPS 80:20 liposomes untreated, treated with Lpt, or with Triton X-100 0.1%. (**D**) Size distribution of PCPS 80:20 liposomes untreated, treated with Lpt-like, or with methanol 4%. Liposomes were incubated for one hour at RT before measurements.

To gain deeper insights into the membrane permeabilization process, we utilized dynamic light scattering (DLS) analysis to track changes in the size of liposomes upon the addition of Lpt or Lpt-like. Figure 4A illustrates that untreated liposomes exhibit a size distribution centered around 110 nm. Upon the addition of Lpt at a concentration of 4 μM (the highest concentration utilized in leakage experiments), followed by a 1 h incubation at room temperature, the liposome size increased slightly to 130 nm, in addition to the appearance of a smaller peak around 5000 nm in diameter. Conversely, treatment with Triton X-100 led to a shift in the primary peak to 8 nm, indicative of micelle formation (as denoted by the yellow line), along with the appearance of a smaller peak centered at 1400 nm, likely attributed to the formation of larger particles. Lpt-like treatment led to similar results with a slight increase in size (Figure 4B). As a control, the addition of methanol without Lpt did not affect the size of liposomes (Figure 4B). Consequently, it can be inferred that the mechanism of action of Lpt peptides involves membrane permeabilization without inducing liposome disruption. The observed slight increase in size likely arises from the interaction between the peptide and potential alterations in the membrane bilayer’s rigidity [31]. The effect of Lpt on the size of liposomes was also analyzed using PCPS (80:20) liposomes. As in the case of PC liposomes, the addition of Lpt or Lpt-like did not change the peak position, while the size distribution became broader, suggesting remodeling of PCPS liposomes in the presence of Lpt peptides (Figure 4C,D). Attempts to use 100 nm PCPS (50:50) LUVs were unsuccessful due to liposome instability (Figure S2).

### 3.5. CD Spectroscopy of Lpt and Lpt-like in the Presence of Liposomes

**Figure 5:**
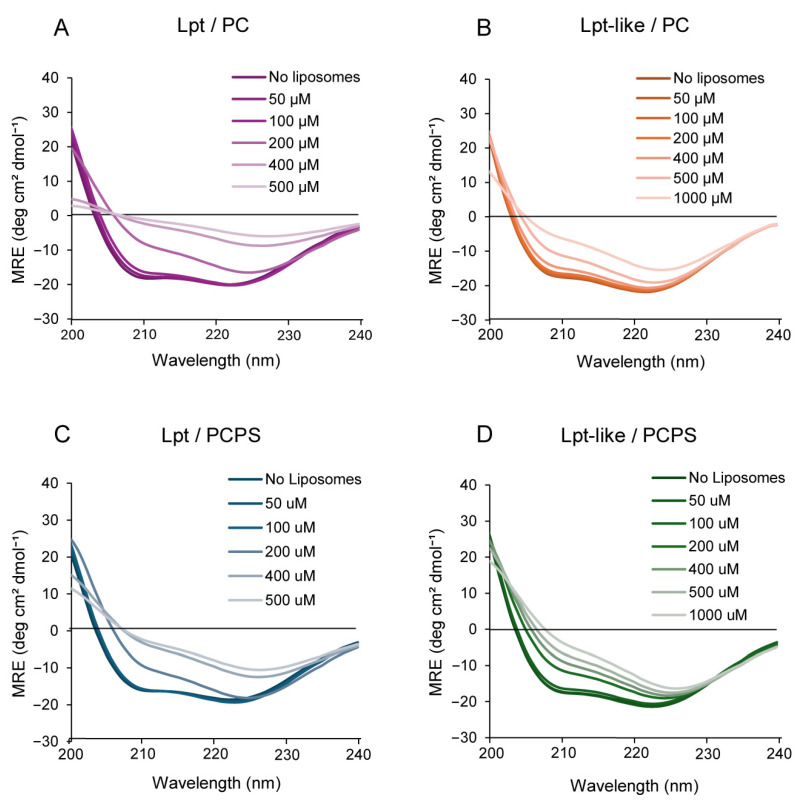
CD spectra of Lpt peptides of 100 μM in phosphate buffer pH 7.4 in the absence and in the presence of increasing concentrations of liposomes. (**A**) Lpt with 100 nm PC liposomes. (**B**) Lpt-like with 100 nm PC liposomes. (**C**) Lpt with 100 nm PCPS liposomes. (**D**) Lpt-like with 100 nm PCPS liposomes.

To understand whether the interaction of Lpt with liposomes alters the secondary structure of the peptides, we have measured the CD spectra of a solution containing 100 μM Lpt or Lpt-like in phosphate buffer at increasing concentrations of 100 nm PC or PCPS liposomes (for simplicity, we express the liposome content in terms of PC or PCPS concentration). As shown in Figure 5A, the addition of liposomes up to a concentration of 100 μM PC does not significantly change the CD spectra of Lpt. However, at higher PC concentration, the characteristic minima at 208 and 222 nm become less pronounced until they disappear at a PC concentration of 500 μM. This result indicates that the interaction of Lpt with the liposomes causes loss of the α-helical structure. A similar behavior was observed also with Lpt-like, although, in this case, the α-helix seems more stable as the two minima do not completely disappear, even in the presence of a concentration of 1 mM PC (Figure 5B). In the case of PCPS, Lpt shows a behavior like that observed with PC, while Lpt-like appears less stable, as the denaturation began at a lower concentration of liposomes (Figure 5C,D).

To verify that the change in CD spectra is not due to light scattering of the liposomes, we have measured the CD spectra of DksA (a small α-helical protein) under the same conditions. As shown in Figure S3, in the presence of 500 μM or 1 mM liposomes, the typical α-helix minima are only slightly affected.

## 4. Discussion

To investigate how Lpt toxins, part of the Lpt/RNA type I TA systems, interact with membranes, we conducted in vitro assays using both Lpt and Lpt-like peptides alongside synthetic lipid vesicles. Prior to these assays, we confirmed the toxic activity of Lpt-like, a 29-amino-acid peptide with a sequence similarity of 69% to Lpt, in vivo using *E. coli* cells. Our data demonstrated that the expression of Lpt-like in *E. coli* induced growth arrest, nucleoid condensation, and cell membrane damage, suggesting that Lpt-like functions as a toxic peptide in *E. coli* by interacting with the cell membrane with effects similar to those of the Lpt toxin [17].

Subsequently, we examined the interaction between Lpt and Lpt-like peptides with synthetic lipid vesicles of different sizes and lipid composition. Leakage experiments carried out by using a fluorescence probe entrapped inside PC liposomes showed that the addition of both Lpt and Lpt-like peptides altered the permeability of the vesicles, inducing the release of the probe on a time scale of 20–30 min. CF release is not determined either by vesicle disruption or fusion, as DLS measurements show only a slight increase in vesicle size upon treatment with both peptides. Furthermore, CF release shows a rapid initial burst of leakage, followed by a very slow release, which does not reach completeness within an hour, even at higher peptide concentrations. This behavior of CF release is inconsistent with the formation of membrane-spanning peptide pores such as those proposed by the barrel-stave model, in which the peptides are aligned and interact laterally. In fact, pore-forming peptides induce a rapid and continuous leakage that persists until all vesicle contents have been completely released [2]. According to calculated release rates, the complete release of a probe from LUV with a single water-filled peptide pore should occur within a few tenths of a second [7]. Moreover, Lpt peptides do not form amphipathic α-helices (Figure 1D) typical of membrane-spanning barrels and their α-helical structure disappears in the presence of liposomes (Figure 5), contrary to what occurs with channel-forming peptides, whose α-helical structure is stabilized in the presence of liposomes.

Conversely, these results suggest a membrane permeabilization mechanism relying on the transient failure of the lipid bilayer (“lipid pores”) as proposed by the interfacial activity model. In this model, membrane permeabilization is driven by a disequilibrium state resulting from the imbalance of charges and surface tension upon peptide binding to the vesicles outer layer [7]. Once the trans-bilayer equilibrium of the peptide is reached, leakage terminates. In this regard, the structure of the Lpt peptides has features that agree with this model; both Lpt and Lpt-like exhibit imperfect segregation of polar and nonpolar residues (Figure 1D) and the central hydrophobic core is constrained by the presence of charged residues (KRDE) at the N- and C-termini. In a previous study [17], we showed that removal of the positively charged carboxy-terminal region of Lpt resulted in a nontoxic peptide as well as changing the amphipathic character of the peptide through the insertion of a charged amino acid into the hydrophobic α-helical segment (P11E). In contrast, mutants obtained by replacing the conserved proline 11 with hydrophobic residues (P11A and P11V) retained the same toxicity as the wt peptide. Although it is difficult to make a correlation between the in vitro experiments and the in vivo observations, it is tempting to speculate that the growth arrest of *E. coli* cultures observed after peptide induction and the subsequent growth restart are determined by a transient membrane failure as proposed by the interfacial activity model [7].

With 100 nm PC liposomes, Lpt was more effective than Lpt-like. Despite the difficulty in correlating the permeabilization activity with the amino acid composition and structure of the peptides, the different physical–chemical properties of the two peptides, determined by the distribution of charged residues at the N- and C-termini, account for their differing behaviors. The calculated isoelectric points further differentiate these peptides, with Lpt and Lpt-like having values of 8.21 and 5.88, respectively. Interestingly, both peptides were significantly less active with 1 μm PC liposomes, indicating that membrane permeabilization is highly affected by the membrane curvature strain.

To evaluate the contribution of electrostatic interactions in peptide–membrane interaction, Lpt was also analyzed with negatively charged PCPS 80:20 liposomes. In this case, the rate of leakage increases significantly at all concentrations, as shown by the τ values. This could be attributed to the increased attraction exerted by the negatively charged liposome surface toward the peptide.

Interestingly, CD spectroscopy analyses showed that both Lpt and Lpt-like peptides lost their α-helical secondary structure after incubation with increasing concentrations of liposomes. This effect was more pronounced for Lpt compared to Lpt-like, in accordance with the higher thermal stability of the latter. This behavior is opposite to what has been reported in the literature for most membrane-interacting peptides, where interaction with lipids typically leads to the stabilization of the α-helical structure [32,33]. However, in a few cases, the interaction of a structured peptide with the membrane resulted in the loss of its native structure [34,35].

## Data Availability

Dataset available on request from the authors.

## Supplementary Materials

The following supporting information can be downloaded at: https://www.mdpi.com/article/10.3390/biom14080994/s1, Figure S1: DLS size distribution of 1 μm PC liposomes; Figure S2: DLS size distribution of PCPS 50:50 after three day of incubation 4 °C; Figure S3: CD spectra of DksA.
