# Supplementary material for: Activity of Membrane-Permeabilizing Lpt Peptides
Source: Biomolecules. 2024 Aug 13;14(8):994. doi: 10.3390/biom14080994 (PMC11352940; doi:10.3390/biom14080994)
Supplement: Supplementary file 1 [file biomolecules-14-00994-s001.zip › biomolecules-3111492-supplementary.pdf]

Activity of Membrane-Permeabilizing Lpt Peptides

Supplementary Materials

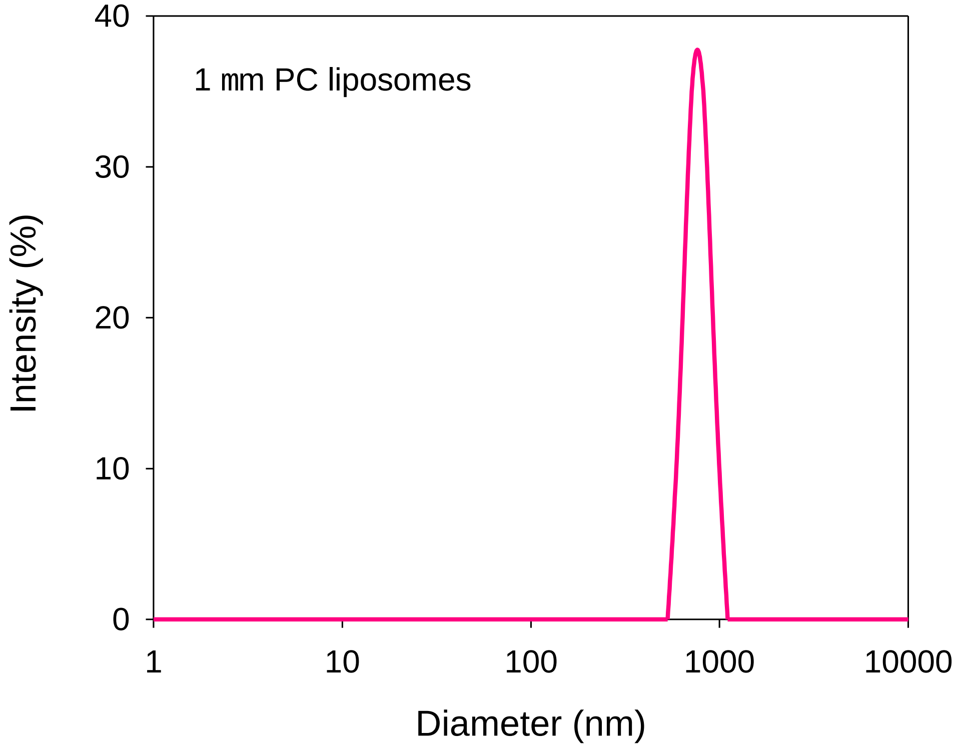

Figure S1. DLS size distribution of 1 μm PC liposomes.

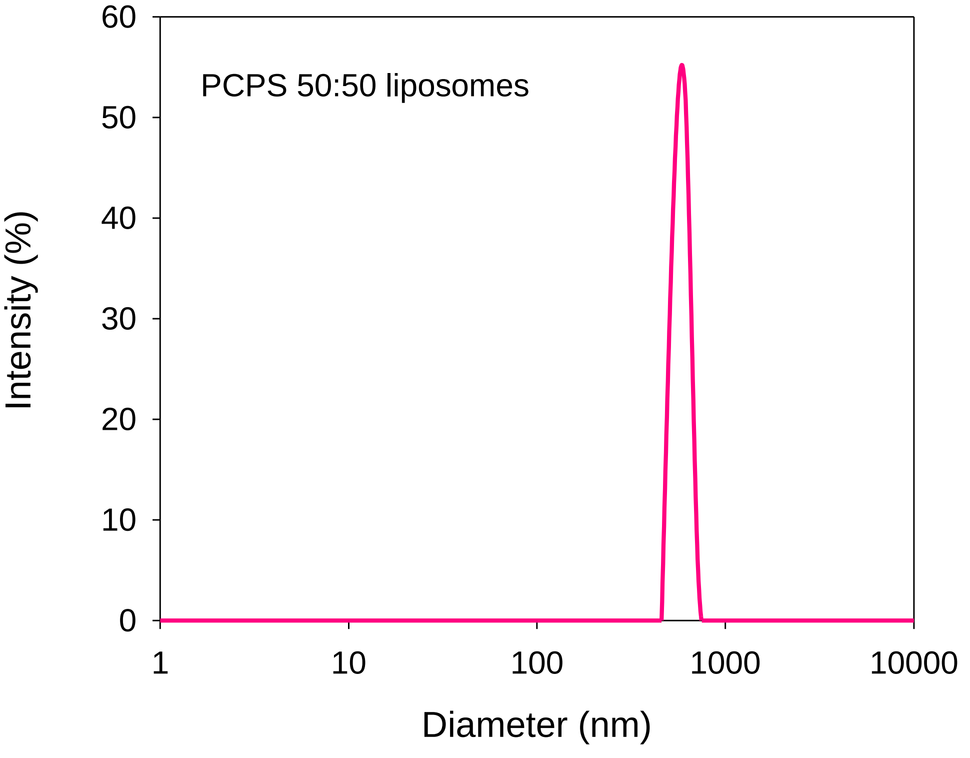

Figure S2. DLS size distribution of PCPS 50:50 after three day of incubation 4 °C.

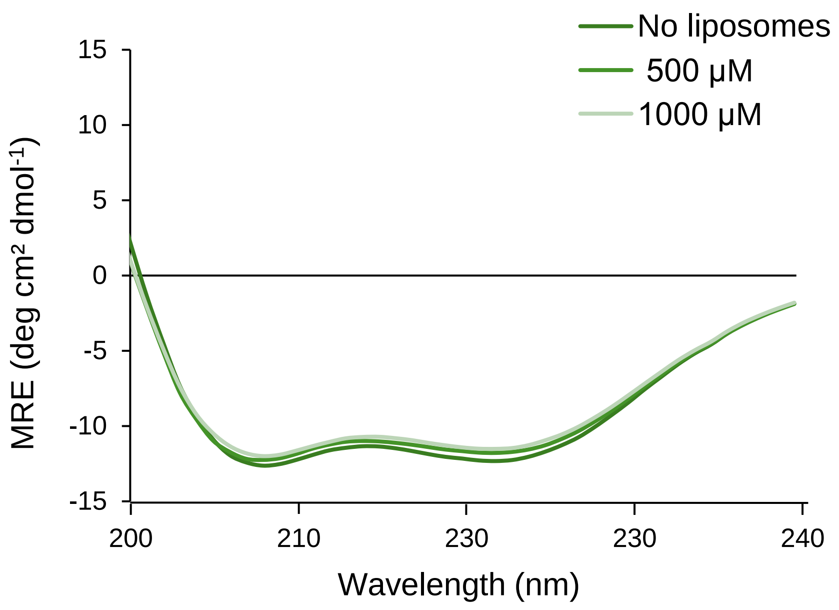

Figure S3. CD spectra of DksA (a small alpha helical protein) 20 μM in phosphate buffer pH 7.4 in the absence and in the presence of 100 nm PC liposomes.
